# Friction reduction through biologically inspired scale-like laser surface textures

**DOI:** 10.3762/bjnano.9.238

**Published:** 2018-09-26

**Authors:** Johannes Schneider, Vergil Djamiykov, Christian Greiner

**Affiliations:** 1 Institute for Applied Materials (IAM), Karlsruhe Institute of Technology (KIT), Kaiserstrasse 12, 76131 Karlsruhe, Germany; 2 KIT IAM-CMS MikroTribologie Centrum µTC, Strasse am Forum 5, 76131 Karlsruhe, Germany

**Keywords:** bioinspiration, friction, laser surface texturing, scales, tribology

## Abstract

Reducing friction forces is a major challenge in many engineering applications involving moving parts. For the past 50 years, the morphological texturing of surfaces for improving tribological properties has been investigated. Only recently, the application of biologically inspired surface features, like scales found on lizards and snakes, has come to the attention of tribologists. Here, we present results of the lubricated and unlubricated performance of biologically inspired scale-like textures applied with laser light to the surface of bearing steel pins. These were paired in unidirectional sliding against metallic (100Cr6), polymeric (PEEK) and ceramic (Al_2_O_3_) counter bodies. Additionally, a possible size effect was investigated by changing the scale diameter between 13 and 150 µm under dry sliding contact against sapphire. Our results demonstrate that depending on the contact conditions a biologically inspired surface morphology has the potential to reduce friction forces by more than 80%. However, under certain conditions, especially for slow-moving lubricated steel-on-steel and steel-on-ceramic contacts, these surface morphologies may increase friction as well. Similar to classical laser surface textures, such as round dimples, these biologically inspired morphologies need to be carefully optimized for each tribological system in which they are intended to be applied. There is no standard solution for all sliding conditions. The results presented here demonstrate that such efforts have the potential to yield significant reduction in friction forces and are expected to spark future research in the field of biologically inspired surface morphologies applied to tribological contacts.

## Introduction

Friction and wear are responsible for more than 20% of the world’s total energy consumption [1]. This staggering number demonstrates that tribology, the study of interacting surfaces in relative motion, is a prime candidate when it comes to reducing CO_2_ emissions and discovering a more efficient use of resources. In order to do so, new strategies for optimized tribological systems have to be considered. Among them are the formulation of new lubricants [2–3], novel coatings [4–6], a more thorough understanding of the basic materials science principles governing friction and wear of bulk materials [7–9], and the morphological texturing of the sliding surfaces themselves [10–11]. The latter has attracted a lot of attention in the last two decades, even though first experiments demonstrating the potential of a morphological surface texture go back to the 1960s [11]. Among the different approaches to realize such textured surfaces, such as chemical etching [12] or material indentation [13], the use of lasers is established as the most versatile and economical method [14]. The most common texturing element is the round dimple [10,14], with which friction reduction of around 80% has been achieved for mixed lubrication contacts [15–16].

While these traditional texturing elements have been studied for decades and by numerous research groups worldwide, in recent years, a new paradigm has emerged. Researchers have started to look to biology in search for morphological textures that would allow for tribologically optimized surfaces [17]. Among the animals and biological structures that have been considered are butterfly wings [18], beetles and earthworms [19], scorpions [20] as well as (and most importantly) the skin of snakes and sand fish lizards [21–24]. It has been demonstrated, for example, that sandfish skin exhibits low friction and little wear [25–26]. The development of manufactured surface textures that are inspired by animals with scale-like surface morphology has resulted in fascinating insights. For texturing a titanium alloy, a lithography-based method was chosen that resulted in regular, elliptical protrusions, lowering friction compared to an untextured control sample [24]. In an approach resulting in surface textures much closer to the natural example, Baum et al. showed that variations in the effective elastic modulus (therefore allowing for damping effects) have a significant influence on the occurrence of stick–slip motion in biological systems and manufactured structures [21]. Baum et al. additionally focused on investigating the microstructure within the scales and aspects of mechanical interlocking between them. The same group of authors convincingly demonstrated frictional anisotropy and a maximum friction reduction of about 50% with biologically inspired surfaces [22]. These investigations relied on a two-step moulding technique for manufacturing scale-like structures and therefore focused on the frictional properties of polymeric samples.

In contrast, our own previous work aimed at retaining the advantages of using laser light to generate a morphological scale-like surface texture [23]. We successfully textured bearing steel surfaces with scale-like morphological patterns and were able to reduce dry friction forces by more than 40% when sliding against a ceramic material, i.e., sapphire [23]. At the same time, these morphological surface textures significantly increased friction for a lubricated contact, seriously impeding their practical applicability in engineering components. Experiments for dimpled surfaces clearly demonstrate the existence of size effects under mixed lubrication [16,27] as well as for the transition from static to dynamic friction in dry contacts [28]. This paper presents results for two independent sets of experiments aiming at investigating the existence of a similar size effect for scale-like structures and at improving the usefulness of such a morphology under lubricated conditions: The first set of experiments applies a constant scale-like morphology to bearing steel surfaces which are tested in dry and lubricated sliding against metallic, polymeric and ceramic counter bodies for a variation of sliding speeds. The second set of experiments aims at investigating a possible size effect under dry sliding against sapphire for a constant sliding speed by varying the scale size by more than a factor of ten.

## Results and Discussion

### Laser surface texturing

**Figure 1:**
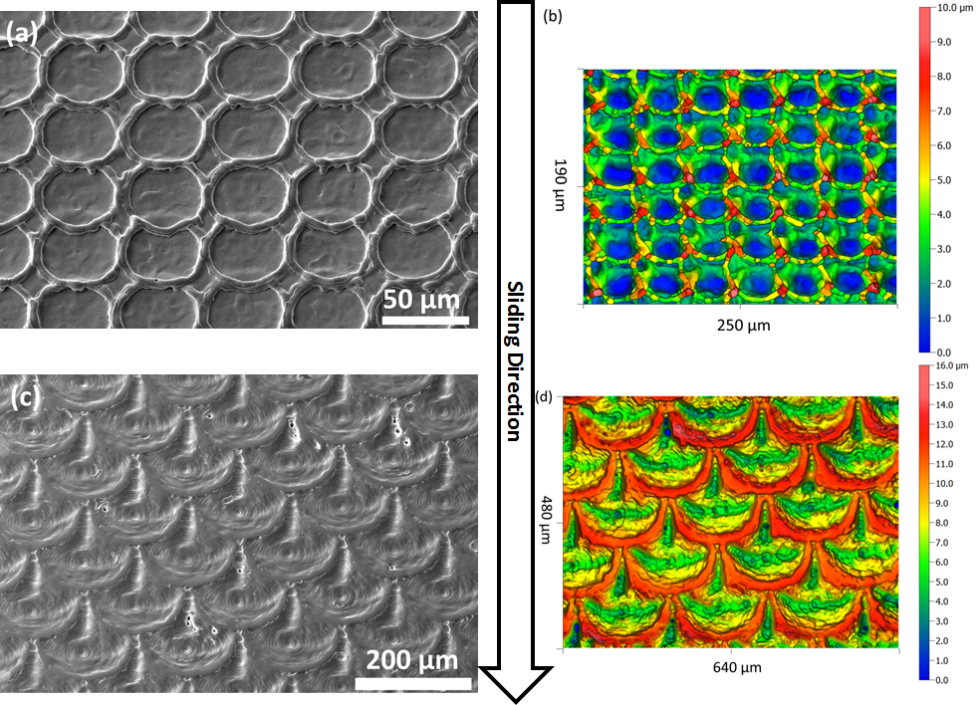
Scanning electron microscopy and white light profilometry images of laser-textured bearing steel (100Cr6) surfaces with biologically inspired scale-like surface morphologies. (a,b) A linear arrangement in parallel rows as tested under dry and lubricated sliding against metallic, polymeric and ceramic counter bodies. (c,d) Arrangement with an offset. The secondary electron SEM images in (a,c) were taken under a tilt angle of 30°. The sliding direction for the textured pins is indicated by the arrow.

In order to investigate the tribological performance of biologically inspired scale-like surface textures in dry and lubricated contacts against metallic, polymeric and ceramic counter bodies, bearing steel pins were laser-textured. The texturing elements chosen were scale-like structures organized in parallel rows with a 100% coverage of the surface (dimple diameter ≈30 µm, height ≈5 µm). Figure 1 presents scanning electron microscopy (SEM) and white light profilometry results for these textures (Figure 1a and 1b), demonstrating that such samples were successfully fabricated with high reproducibility for the scale-like texturing elements.

For the testing of a possible size effect under dry sliding conditions, a different scale-like surface morphology was chosen. Here, the scales were organized with an offset between each row, resulting in a surface texture as presented in Figure 1c and 1d. This surface morphology was created for four different scale sizes between 13 and 150 µm in scale diameter (measured in vertical direction). The height of all the scale-like structures was 6 ± 1 µm, independent of scale organization and size.

### Variation in counter body material

#### Metallic counter body (100Cr6)

**Figure 2:**
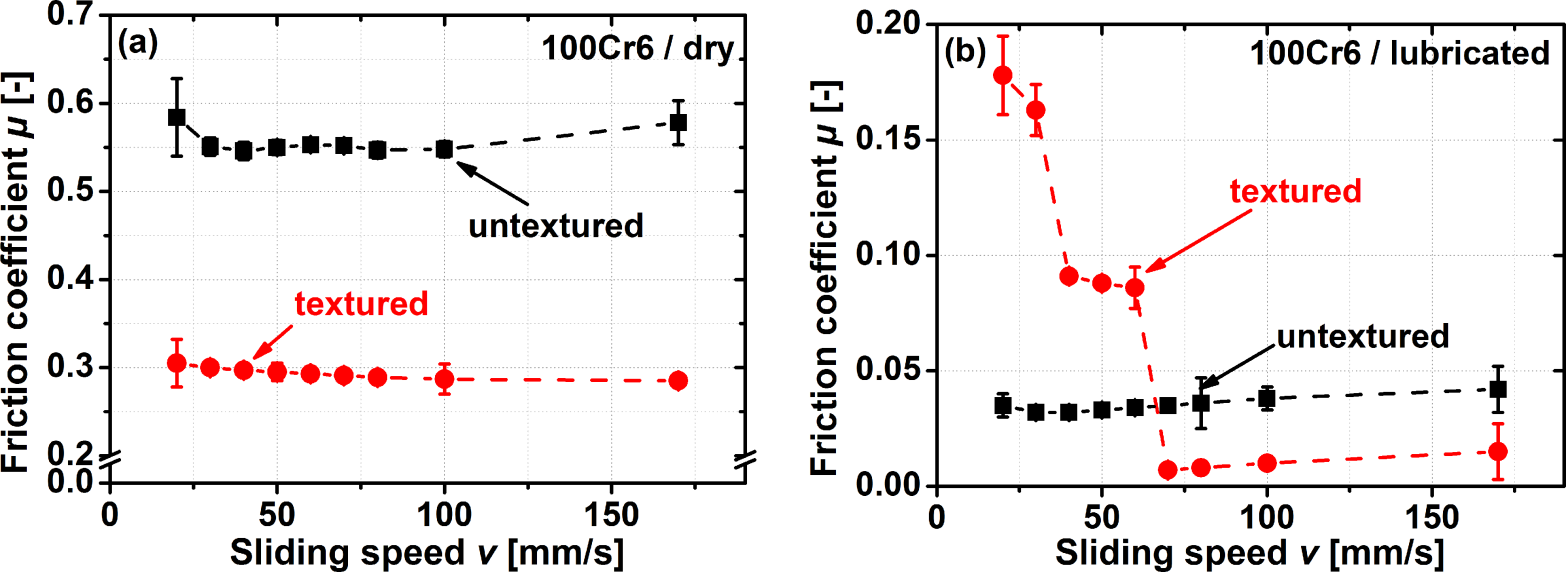
Stribeck curve-like pin-on-disc experiments for a variation in sliding speed from 20 to 170 mm/s for dry (a) and lubricated (b) steel-on-steel contacts. The normal force was 2 N during all experiments.

For testing the friction performance of this laser-generated biologically inspired scale-like surface morphology in contact with a metallic counter body, one of the most common bearing steels (100Cr6) was chosen; the same steel that the pin is made out of. The results of Stribeck curve-type experiments for dry and lubricated sliding conditions for textured samples as well as for untextured controls are presented in Figure 2.

For the dry sliding experiments presented in Figure 2a, the sliding speed did not have a significant influence on the resulting friction coefficient. For the textured samples, a minimal decrease in the friction coefficient (≈9%) with sliding speed was measured. Through the introduction of a scale-like surface texture, a reduction in the friction coefficient over the entire range of sliding speeds by approximately 50% was achieved. The resulting friction coefficient of around 0.3 is a notably low value for an unlubricated steel-on-steel self-pairing. Upon lubricating the contact, the results change dramatically. The absolute values of the friction coefficients decrease by almost one order of magnitude, and even more importantly, the efficiency of adding the surface texture now strongly depends on the sliding speed. In the untextured case, roughly the same friction coefficient (µ ≈ 0.04) was measured over the entire range of sliding speeds. For the scale-like structures, the friction coefficient at a sliding speed of 20 mm/s is 0.18, decreasing sharply when increasing the sliding speed to 70 mm/s down to a value of µ = 0.007. This corresponds to a more than 60% decrease in friction. Upon further increase in the sliding speed up to the maximum speed tested (170 mm/s), the coefficient of friction doubles to 0.015. When comparing the textured and untextured samples, the textured ones demonstrate a more classical Stribeck curve-like behaviour, whereas the untextured controls do not demonstrate a significant influence of the sliding speed. As a result, the potential for decreasing friction forces by introducing a scale-like surface morphology in lubricated contacts strongly depends on the sliding speed. For sliding speeds below 70 mm/s, surface texturing results in an increase in the friction coefficient. In the case of the smallest sliding speed tested (20 mm/s), this increase was by more than a factor of five. This changes for sliding speeds above 70 mm/s, where introducing a scale-like surface texture decreases friction coefficients by about a factor of four. This is in the range of the 80% friction reduction that was previously reported in lubricated steel-on-steel contacts for surfaces textured with round dimples (the most common pattern reported in laser surface texturing) [15–16]. These results signify a substantial improvement over the friction behaviour of scale-like laser surface textures in lubricated contacts, where friction forces always increased by up to a factor of three [23]. This demonstrates that, at least over a certain range of sliding speeds, these biologically inspired surface morphologies have the same potential for reducing friction forces as standard surface textures, which have already been optimized for several decades [10].

The plateau in Figure 2b for sliding speeds between 40 and 70 mm/s may stem from different frictional interactions, or by run-in effects. Running-in is a difficult subject to understand [29–30] and it is known that the sliding speed can have a deciding influence on run-in [31–34]. Similar effects may in part explain the phenomena illustrated in Figure 2b. Answering this question will require run-in experiments, e.g., by probing the subsurface microstructure evolution.

Another way to understand why these textures demonstrate a non-typical Stribeck curve behaviour for the lubricated experiments might be found in the fact that, compared to classical surface textures, the debris formed during laser texturing was not removed. In fact the scale-like structures may be viewed as the debris. Therefore, the minimum thickness for the oil film to fully separate the two contacting bodies might need to be higher compared to round dimples. One can argue that with reducing sliding speed, solid–solid interactions occur earlier, explaining the first plateau in friction forces encountered for sliding speeds between 40 and 70 mm/s. For even slower sliding, a more classical mixed lubrication contact is formed, as it is also encountered for samples textured with dimples [15].

The surface roughness of the steel discs after the experiments was *R*_a_ ≈ 200 nm. The discs therefore became slightly rougher during the experiments. At the same time, surface profilometry did not reveal any major scratches or signs of wear.

#### Polymeric counter body (PEEK)

**Figure 3:**
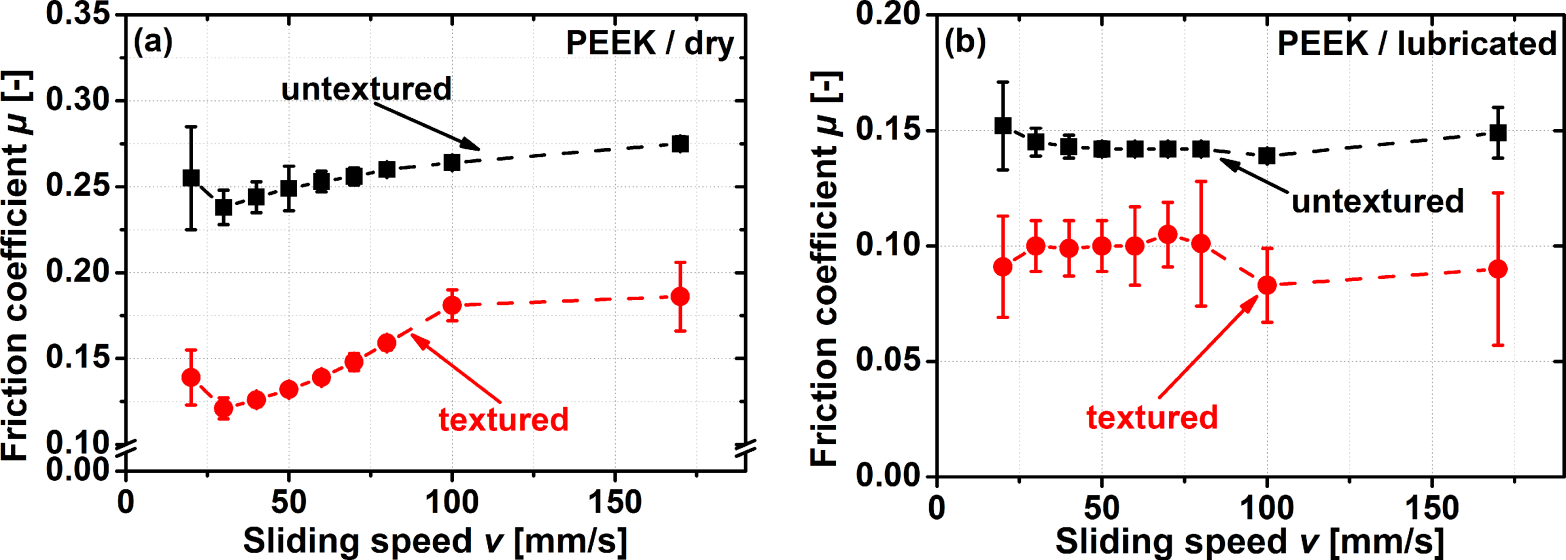
Stribeck curve-like pin-on-disc experiments for a variation in sliding speed from 20 to 170 mm/s for dry (a) and lubricated (b) steel-on-polymer contacts. The normal force was 2 N during all experiments.

Polyetheretherketone (PEEK) is a polymer widely employed in tribological applications, and therefore an excellent choice for testing the frictional properties of a laser-generated, biologically inspired, scale-like surface morphology when paired against a polymeric counter body. The results for experiments performed with and without lubrication are presented in Figure 3.

In a tribological contact without lubrication (Figure 3a), both for the textured sample, as well as for the untextured control, the friction coefficient shows the same qualitative trend with the sliding speed. A small decrease between the first two sliding speeds tested, followed by an increase in friction coefficient up to a sliding speed of 100 mm/s and then a significantly smaller increase when the sliding speed reaches 170 mm/s. As these are the results for an unlubricated contact, it has to be pointed out that the trends observed in this diagram cannot be explained by the transition from mixed to hydrodynamic lubrication. For all sliding speeds tested, texturing the sample resulted in a significant decrease in the friction coefficient between 30% (*v* = 100 mm/s) and 50% (*v* = 30 mm/s).

When lubricating the contact (Figure 3b), the friction forces decreased compared to dry sliding conditions, but significantly less compared to the steel-on steel-contact presented in Figure 2. For the polymeric counter body investigated here, the scale-like surface morphology reduced friction over the entire range of sliding speeds tested. The efficiency of laser surface texturing depends on the sliding speed and the reduction in the friction coefficient varied between 20–30% for speeds between 20 to 80 mm/s and by more than 40% for speeds between 100 to 170 mm/s. The maximum reduction in the friction coefficient for the steel-on-polymer contact therefore is significant, but less compared to the steel-on-steel one. No transition from a beneficial to a detrimental effect by introducing a scale-like surface morphology for smaller sliding speeds was observed.

Surface profilometry on the discs after the experiments revealed surface roughness values of *R*_a_ ≈ 250 nm, demonstrating that the discs became slightly rougher. Occasional signs of adhesive wear events were observed (see Figure S3, Supporting Information File 1 for an optical micrograph of a PEEK disc after a tribological experiment).

#### Ceramic counter body (Al_2_O_3_)

**Figure 4:**
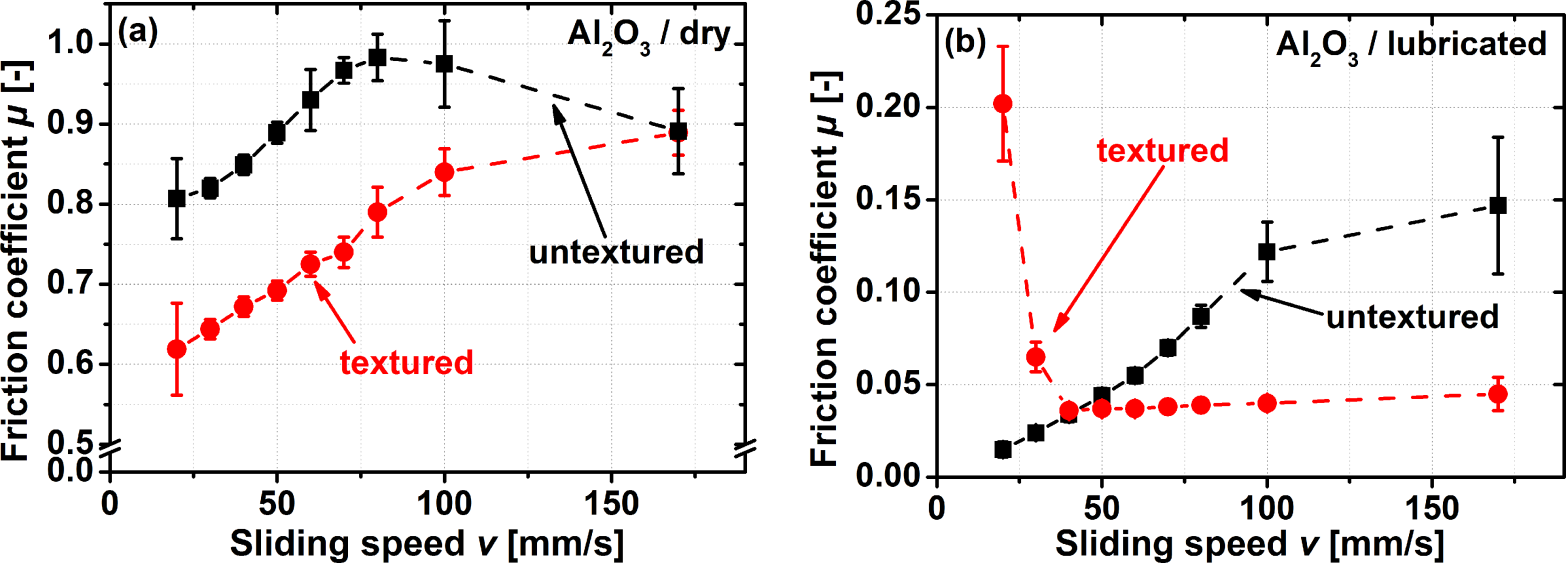
Stribeck curve-like pin-on-disc experiments for a variation in sliding speed from 20 to 170 mm/s for dry (a) and lubricated (b) steel-on-ceramic contacts. The normal force was 2 N during all experiments.

For tribological applications, a wide range of ceramics has been experimentally tested in contacts involving laser surface textures. Aluminium oxide is a common material in those investigations [35–36]. Therefore, and because Al_2_O_3_ can be considered as the model engineering ceramic, it was chosen as the counter body material for testing the performance of the scale-like surface morphology in a steel-on-ceramic contact. The results of these experiments for dry and lubricated sliding are presented in Figure 4.

For dry sliding (Figure 4a), the textured and the untextured samples demonstrate the same qualitative trend of an increasing friction coefficient with sliding speed. The average friction reduction for sliding speeds between 20 to 80 mm/s is 21%. For a sliding speed above 80 mm/s, the friction coefficient for the textured sample continues to increase, whereas for the untextured control, it decreases. These opposite trends converge at the same value for the friction coefficient (µ = 0.89) for the textured and untextured samples at a sliding speed of 170 mm/s. This suggests that for an unlubricated steel-on-ceramic contact, texturing the steel surface with a scale-like morphology is beneficial only for low sliding speeds, and might be detrimental for higher ones. It should be noted that the absolute values for the friction coefficient for the steel-on-ceramic contact are significantly larger compared to the steel and polymer counter bodies presented above. This might be due to the significantly larger surface roughness of the aluminium oxide discs compared to the PEEK and 100Cr6 ones. Unfortunately, it is experimentally very challenging to prepare all three counter body materials to the same surface roughness. When testing the dry friction performance of biologically inspired surface morphologies, Baum et al. [22] reported the opposite effect of decreasing friction with surface roughness. This was explained with a decrease in apparent contact area when roughness increased. We did not observe this effect in our data, but it should be noted that we did not systematically change the roughness of one contacting material. The Young’s modulus of the ceramic and the steel making up the contact tested here is larger than the Young’s modulus of the epoxy resin and the glass ball that were paired by Baum et al. [22]. This could be another factor explaining this apparent difference.

For the lubricated contact, the frictional behaviour is similar compared to what was discussed above for the steel-on-steel contact. The efficiency of laser surface texturing strongly depends on the sliding speed regime. For sliding speeds below 40 mm/s, the scale-like surface morphology increased the friction coefficient; for sliding at 20 mm/s, it increased by more than a factor of 13. This might again be explained by the fact that once the thickness of the oil film is no longer sufficient to fully separate both bodies, solid–solid contacts are formed. Especially with a brittle, stiff ceramic, such contacts are expected to result in a very pronounced friction increase compared to the hydrodynamic regime. This is exactly the behaviour visualized in Figure 4b. For sliding speeds above 40 mm/s, the biologically inspired surface morphology decreased the friction coefficient. The fact that the friction increased for the textured surfaces when pairing them in slow moving, lubricated contacts against ceramics or metals might additionally be explained by the minimal influence of (micro) hydrodynamic effects at low sliding speeds. Together with the high hardness and Young’s modulus of Al_2_O_3_ and 100Cr6, this probably results in a small true contact area and therefore large effective contact pressures. These effects manifest themselves in friction forces that are closer to what one might expect for unlubricated contacts. The efficiency of laser texturing increases with sliding speed, reaching its maximum of 70% for a speed of 170 mm/s. For faster sliding, lubricated contacts, the scale-like surface texture therefore allows for a significant reduction in friction forces, similar to what has been reported for the maximum efficiency of adding round dimples to steel-on-ceramic contacts [37].

The surface roughness of the Al_2_O_3_ discs was measured to be *R*_a_ ≈ 590 nm, demonstrating that the ceramic counter bodies slightly flattened during the tribological experiments. No signs of wear were detected by optical profilometry.

#### Size effect under dry sliding

**Figure 5:**
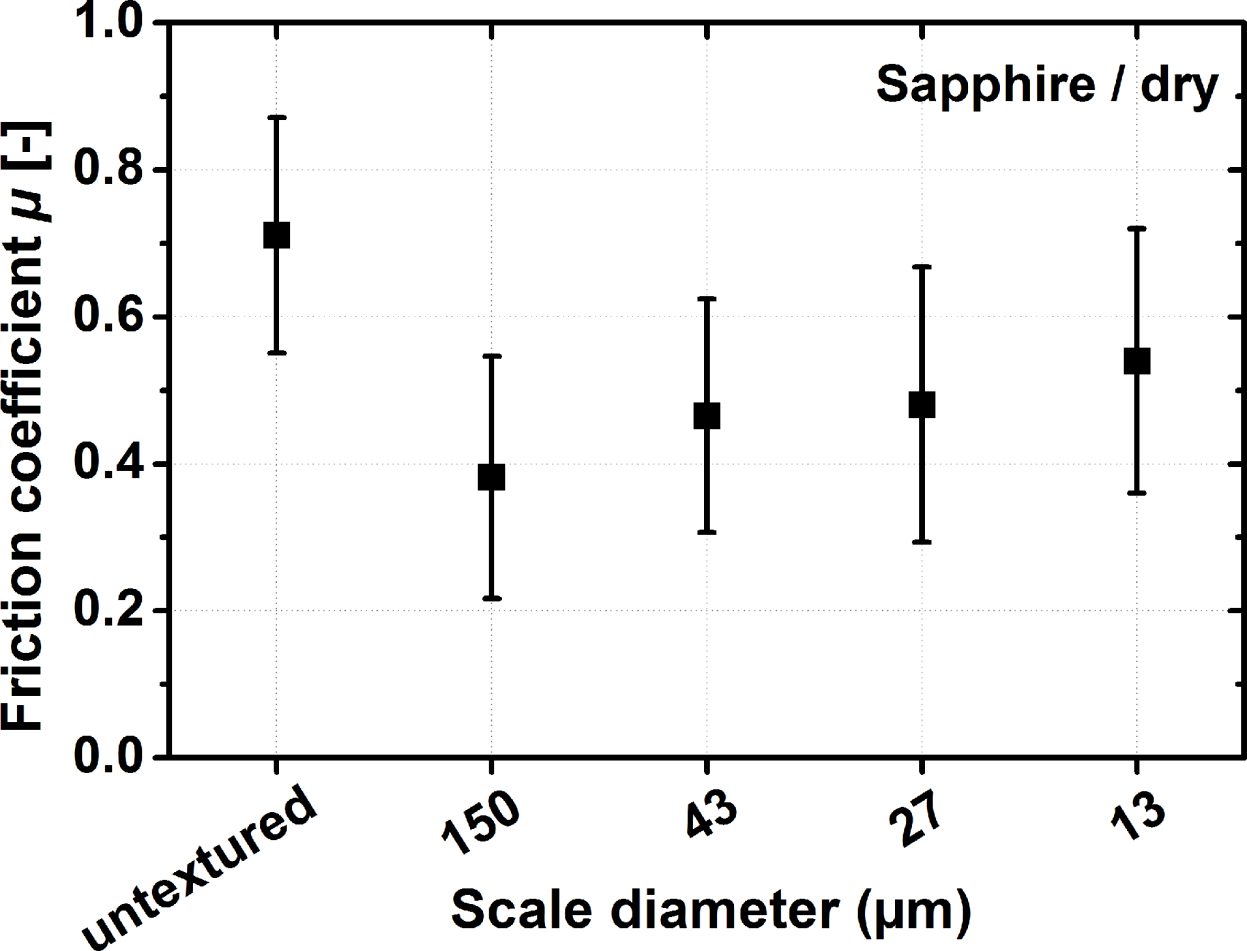
Comparison of the average friction coefﬁcient for the last 250 m of unlubricated tribological experiments for a total sliding distance of 1000 m. Results are presented for untextured polished controls and scale-like surface morphologies with scale diameters between 13 and 150 µm. The experiments were conducted without lubrication and a steel-on-ceramic contact with sapphire as counter body material. The normal force was 2 N and the sliding speed 100 mm/s during all experiments. Please note that the abscissa is not to scale.

When testing the tribological performance of round dimples created by laser surface texturing, considerable size effects for dry and lubricated experiments, as well as for static and dynamic friction were reported [15–16,27–28]. This is true for the dimple diameter [15,27–28], as well as for the dimple depth [16,28]. Size effects are a common phenomenon in materials science and have been found to influence mechanical and magnetic as well as surface properties like adhesion [38–41]. We therefore experimentally investigated whether such an effect might also exist for the friction properties of a laser-generated biologically inspired scale-like surface morphology. For doing so, we systematically varied the diameter of scale-like structures between 13 and 150 µm. In contrast to the textures investigated against different counter bodies presented above, the ones tested here were organized with an offset (see Figure 1c and 1d). The reason for choosing this pattern compared to the ones employed when investigating the effect of different counter bodies is that the offset scales can be manufactured with higher precision with respect to the scale diameter over a larger range in diameters compared to the ones tested above. Additionally, we wanted to probe the existence of such a size effect against one of the most well defined counter body materials available: polished sapphire discs. These experiments concentrated on dry sliding conditions with the results being presented as the average friction coefficient for the last 250 m of each experiment in Figure 5.

The results in Figure 5 clearly demonstrate that a size effect exists in dry sliding. When increasing the dimple size from around 10 to 150 µm, the friction coefficient decreased by 30%. Compared to the untextured control, the friction coefficient was reduced by a factor of two for the largest scales tested.

Explaining these results is currently difficult. We attempted to follow the argumentation of Baum et al. who stated that minimal friction is achieved for bioinspired structures when the true contact area is minimized without allowing for mechanical interlocking [22]. When doing so, we neglected that the local contact pressure will increase with decreasing contact area (assuming a constant normal load) and the complication that arguments based on indentation depth do change for a sliding contact (e.g., Hamilton’s [42] instead of Hertz’s [43] solution) should be applied for the stress field. We made use of the contact mechanics solver developed and provided by Pastewka [44]. This program allows uploading white light profilometry images and analysing the contact mechanics. Profilometry images of all four scale sizes were taken and attention was focused on the ratio of the true to the projected contact area for a contact with a sapphire counter body. This analysis showed that, in agreement with what was postulated by Baum et al., the true contact decreased for the smallest to the largest scale size by about a factor of three.

Size and scaling effects in biologically inspired surface textures are well known to exists, for example, in fibrillary adhesives inspired by the hierarchical hairy structures found on the feet of certain lizards and insects [39–40,45].

There are four effects that are classically used to argue why laser surface texturing has a beneficial influence on tribological properties – the trapping of wear debris [46], changes in the contact angle [47], the storage of lubricant [48] and an additional micro-hydrodynamic pressure build-up effect [49] – however, these seem not to be able to explain our results presented here. Wear is negligible in our experiments (see below), changes in contact angle between the differently textured surfaces are expected to be minimal, and there cannot be any storage of lubricant or micro-hydrodynamic pressure build-up as these experiments were performed under dry sliding. Understanding and exploiting this size effect phenomenon therefore will be the focus of future research. These investigations will mainly involve a detailed modelling of the contact mechanics for each surface morphology. We will also investigate whether even larger scale-like structures further decrease friction, or upon which limit in scale size the friction coefficient increases again. It will also need to be tested whether a similar size effect exists for lubricated contacts and for counter body materials other than sapphire.

Attempting a holistic examination of all of the results presented above, it becomes apparent that the texturing effect on friction of a bearing steel surface with biologically inspired scale-like surface morphology strongly depends on the exact circumstances of the sliding contact. This is in agreement with what was reported for polymeric bioinspired surface morphologies [22]. Among the factors that need to be considered are: contact lubrication conditions, sliding speed and the counter body material. Certainly, future experiments will reveal that even more parameters need to be considered, for example, reciprocating versus unidirectional sliding, temperature, and the viscosity of the lubricant. As with dimpled laser surface textures, and also for biologically inspired ones, there is no simple “one size fits all” solution. For each tribological application, the surface textures must be optimized individually. At the same time, our results strongly suggest that bioinspired morphologies have at least the same potential for reducing the friction coefficient as standard laser-generated surface morphologies, i.e., by about 80%. Taking into account that the latter ones have been optimized for decades and the scale-like surface textures only recently have emerged as a research focus, it is quite likely that their full potential for positively influencing tribological properties has not yet been reached. This is especially true as wear was not the focus of this study, but will be investigated in more detail in the future. To demonstrate that these scale-like surface textures show only minimal wear under harsh tribological conditions (unlubricated contact, sapphire counter body), an optical micrograph of a sample surface after 1000 m of sliding is presented in Figure S1, Supporting Information File 1. This image demonstrates that wear of these surface morphologies is negligible, in agreement with previous results [23] and with the good antierosion performance of surface textures inspired by scorpion armour [20]. Future research will involve thoroughly quantifying wear rates. In the case of lubricated contacts, we will also involve fluid dynamics modelling to understand and optimize the tribological behaviour of these surfaces. For dimpled surface morphologies, such simulations have recently been able to explain the beneficial effect of laser surface texturing [27,50].

An intriguing result of these simulations is that the depth of the surface textures is expected to have a stronger influence as compared to their lateral size. This result, that was verified experimentally [50], demonstrates that mechanisms explaining classical round dimples can unfortunately not simply be transferred to scale-like surface textures. In our experiments, the variation in depth was below one micrometre. On the contrary, the dimple size has a decisive influence (see Figure 5). These surface textures themselves need to be modelled as realistically as possible, requiring future elaborate fluid dynamics simulations.

As far as the unlubricated experiments are concerned, the concept developed by Bowden and Tabor [51] points out that most frictional energy dissipation is due to plastic deformation of the subsurface layer. If one of the sliding partners is harder than the other, the softer surface will be ploughed to an appreciable depth by the hard surface’s asperities. Thus, the bulk properties of the softer surface determines the friction and wear properties of the entire tribological system [51]. The coefficient of friction in a sliding system accordingly is expressed as the ratio of the shear strength to the yield pressure of the softer metal [51]. Consequently, the tribological properties of the surface are strongly influenced by the subsurface material and the subsurface microstructure is strongly influenced by the plasticity and the nature of the corresponding dislocation activity under a tribological load [8]. The microstructure under the contact therefore undergoes drastic and complex changes during sliding, which in themselves strongly depend on the nature of the tribological loading conditions [52]. We have studied such phenomena in detail recently with high purity copper [53–54] and a pearlitic steel [9] as model materials. Whether and how the microstructure under the sliding contact changes in the tribological systems investigated here will be an interesting task for future research. It is expected that only small microstructural change will be found on the side of the textured bearing steel when it is paired with a much softer material like PEEK, whereas significant changes should be seen when the textured 100Cr6 samples are investigated after being run against alumina. At the same time, the mechanical properties of both contact materials need to be characterized after the experiments to reach a more thorough understanding of where and how frictional energy is dissipated.

When investigating all the counter body materials by optical microscopy and profilometry, no signs of any chemical change (e.g., discoloration) were detected. See, for example, Figure S3, Supporting Information File 1 for an optical micrograph of a PEEK disc after a dry sliding experiment. As described above, only very limited wear was observed, even though not specifically quantified.

When comparing with the literature, it becomes apparent that frictional anisotropy of the laser-generated scale-like surface textures needs to be tested in the future. Such effects were reported for a variety of biological systems [55–56] and polymeric [21–22] as well as metallic [24] surface morphologies inspired by snake skin. Modelling efforts revealed that anisotropy is a function of the counter body’s surface roughness [56]. As the different counter body materials tested here do exhibit a range in roughness, they might be ideal to test similar effects for laser-generated bioinspired morphologies. In contrast to experiments with polymeric scale-like structures tested in frictional loading [55], in our friction data, no sign of stick–slip phenomena were encountered. This discrepancy might be explained by the significantly different contact conditions (e.g., 10 mm sapphire ball vs 1 mm glass sphere) and sliding distances of several hundred meters vs 500 µm. Due to their small Young’s modulus, polymers are known the be more prone to stick–slip effects compared to ceramics and metals. This might be an additional explanation for the absence of stick–slip with these two counter body materials. Compared to our own previous work [23], we were able to significantly decrease friction forces in dry sliding by a further optimization of the scale size and packing strategy. Perhaps even more importantly, friction also decreased upon texturing for lubricated contacts – at least for a wide range of sliding speeds. When testing the dry sliding performance of titanium alloy (Ti6Al4V) pins textured via mask lithography with morphologies inspired by snake skin, Cuervo et al. [24] systematically varied the height, width and spacing of their elliptical surface features. A reduction in friction was also reported for surfaces featuring pillar and channel surface morphologies inspired by biology [57–58]. The authors reported that friction depends on all of these parameters, in agreement with the results presented above for dimple size. We so far did not systematically vary the height of our scale-like structures; a task for future research. It should be noted that compared to mask lithography [24] and double moulding techniques [22], the laser-based texturing applied here is expected to be more flexible for generating different surface morphologies as well as allowing for significantly faster processing times, especially for larger contacting bodies. This will be a crucial factor for applying scale-like surface morphologies in real-life tribological applications, especially as the exact texturing parameters, like the scale size and packing, need to be adapted for each tribological system. The superior results obtained from the application of laser light for the generation of surface morphologies such as dimples is the reason why laser surface texturing has replaced other surface texturing techniques over the last decades [10,15], with notable exceptions such as end milling [59]. Another aspect beckoning future research is the hardness of the scale-like structures created by laser texturing. In biological systems, such as snake skin, it was revealed by nanoindentation that the outer scale layer has a higher hardness compared to the inner ones [60–61]. One could speculate that through the rapid cooling of the molten bearing steel during laser texturing, making up the scale-like morphology, a higher hardness is achieved compared to the underlying material (see the Experimental section).

When texturing surfaces with dimples it was reported that the temperature of the lubricated tribological contact (and thus changes in oil viscosity) have a significant influence on the optimum dimple diameter for maximum friction reduction [15]. A similar effect may exist for the optimum diameter of the scale-like surface morphologies as a function of oil viscosity, something to be tested in future experiments. Among our ongoing work, we have started to test these surface structures in reciprocating contacts, in slurries, and in contacts containing dry, fine oxide powders in order to simulate the environment that animals like sandfish encounter in their natural habitats. Preliminary results indicate that under these conditions, laser-generated scale-like surface morphologies have the potential to significantly lower friction and wear.

## Conclusion

In this contribution, biologically inspired scale-like morphologies were manufactured on bearing steel (100Cr6) surfaces by laser light. When testing these surface textures against metallic, polymeric and ceramic counter bodies under dry and lubricated unidirectional sliding for sliding speeds between 20 and 170 mm/s, we found that:

- For a metallic counter body (100Cr6 steel), dry sliding friction is reduced over the entire range of sliding speeds by about 50%. For lubricated experiments and small sliding speeds below 70 mm/s, friction is larger when applying a surface texture, whereas it decreases upon texturing for faster sliding by up to 80%.
- For a polymeric counter body (PEEK), dry sliding friction is decreased when applying the surface texture by 30–50% for all sliding speeds. For a lubricated contact, friction decreases for the textured samples over the entire range of tested sliding speeds, on average by 40%.
- For a ceramic counter body (Al_2_O_3_), dry sliding friction is decreased upon texturing. The reduction in friction strongly depends on the sliding speed. For slow sliding, friction is reduced by about 20% with this decrease becoming smaller with increasing sliding speed. For the highest speed tested (170 mm/s), the textured and untextured samples show exactly the same friction coefficient. In the lubricated case, the potential to positively influence the frictional behaviour of the contact depends strongly on the sliding speed. For a speed below 40 mm/s, the textured surface results in friction coefficients higher than the polished reference, increasing friction by up to a factor of 13. For faster sliding, friction reduction of up to 70% was found for the textured samples.

The efficiency of generating a scale-like surface morphology with laser light for reducing friction heavily depends on the exact conditions of the tribological system they are applied to. Counter body material, lubrication conditions and sliding speed are among the parameters that need to be taken into account. No single texture will be beneficial under all conditions. The scale morphology needs to be adapted to each tribological system, whereby friction reduction of up to 80% is possible – a dramatic savings in frictional energy losses and well worth this effort.

It was also shown that a size effect for the scale size exists in dry sliding against sapphire. When increasing the scale diameter from 13 to 150 µm, friction decreased by 30%. Compared to the untextured, polished reference, friction was reduced by 50%. This size effect is explained by a decrease in true contact area with increasing scale size, and will be tested for lubricated contacts in future experiments.

Wear was not a focus of the present study; however, preliminary results indicate that wear is significantly reduced through the application of a scale-like surface morphology.

## Experimental

### Materials

The tribological testing for both sets of experiments was conducted with pins made out of 100Cr6 (Fe with 1.5% Cr and 1.0% carbon, AISI 5210) bearing steel. The material was purchased from KGM (Fulda, Germany) in the form of spheres having a diameter of 8 mm and with one side flattened. The flattened side of the spheres was further polished to a diameter of 7.5 mm. This allowed for enough area to perform the laser surface texturing. The last polishing step was performed with a colloidal SiO_2_ suspension (OP-U from Struers, Willich, Germany). The entire sample preparation process yielded a scratch-free surface, having a surface roughness of *R*_a_ < 0.01 µm as determined by optical profilometry (Sensofar Plµ neox, Barcelona, Spain).

As counter bodies for the experiments against metallic, polymeric and ceramic samples, discs with a diameter of 50 mm were used. The first counter body material tested was 100Cr6 bearing steel (the same material as for the pins). It was purchased from EisenSchmitt, Karlsruhe, Germany and heat treated to a hardness of approximately 800 HV1. The steel pins had the same hardness. The Young’s modulus for 100Cr6 is 212 GPa. The steel discs were ground (200 mesh) to a roughness of *R*_a_ = 0.09 ± 0.03 µm as measured by a stylus roughness measurement device. Polyetheretherketon (PEEK), a polymer very often used in tribological applications, was the second material chosen. It was supplied by Arthur Krüger GmbH (Barsbüttel, Germany) and after grinding had a roughness of *R*_a_ = 0.20 ± 0.05 µm. The Young’s modulus for PEEK is 3.6 GPa. The Rockwell hardness was measured as M 105. The third counter body material tested was aluminium oxide (FRIALIT F99,7), purchased from FRIATEC AG (Mannheim, Germany). The roughness of these discs after grinding was *R*_a_ = 0.65 ± 0.1 µm. The Young’s modulus was determined to be 384 GPa and the hardness was measured as 1670 HV1.

For the experiments investigating a possible size effect under dry sliding, sapphire discs were used as counter body material. These were prepared as previously described [23] and had a final roughness of *R*_a_ < 0.01 µm, measured by atomic force microscopy (Park XE7, Suwon, Korea). These sapphire discs had a Knoop hardness of 2200 HK and a Young’s modulus of 350 GPa.

The waviness (2nd order according to DIN 4760) along the frictional radius of all discs was below 1 µm as measured by white light proﬁlometry.

### Laser surface texturing

All laser surface texturing was carried out with a Q-switched ytterbium fibre laser (Piranha II, Acsys Instruments, Kornwestheim, Germany). The laser wavelength was 1064 nm, the spot size 10 µm, the pulse duration 2 µs, the laser power 11 W, pulse frequency 41 kHz, the laser speed 2 m/s, the pulse distance 49 µm and the waiting time between rows was 1 s.

The texturing elements chosen for investigating the influence of different lubrication conditions and counter body materials are scale-like structures with a surface coverage of 100%, organized in parallel rows, resulting in a surface as presented in Figure 1a and 1b. For generating these textures, a pulse distance of 49 µm and a row distance of 40 µm were applied. The scale size for this surface morphology was 30 ± 1 µm and was constant for all lubricated and dry tribological experiments. The experiments investigating a possible size effect were conducted with scales organized with an offset between each row (as presented in Figure 1c and 1d). This surface morphology was created for four different scale sizes: 13, 27, 43 and 150 µm (error in scale size approximately 1 µm). The height of all scale-like structures was 6 ± 1 µm. These sizes for the scale-like structures were chosen based on what is accessible with the laser source available and on previous results [23]. A scale diameter of 13 µm approaches the minimum feature size possible with the Q-switched fibre laser [15]. As we wanted to span at least one order of magnitude in diameter, we chose 150 µm to be the largest one tested.

Based on previous results when texturing a pearlitic steel with round dimples, no microstructural changes within the 100Cr6 samples in the heat affected zone were expected [15]. Figure S2, Supporting Information File 1 shows a scanning electron microscopy (SEM) image of a cross-section through a laser-textured sample prepared by focused ion beam microscopy. The image demonstrates that the microstructure of the material under the textures does not change due to the laser processing. Towards and at the edges of the scale-like textures (i.e., the material that became molten during processing), the microstructure of the material is so fine that it can no longer be resolved by SEM. Transmission electron microscopy would be necessary to reveal the microstructure of these structures. It therefore also stands to reason that the hardness at the scale edges is higher than in the middle of the textures. Cross-sectional hardness measurements, performed with a Fischerscope microhardness tester (Helmut Fischer GmbH, Sindelfingen, Germany), revealed a local hardness at the scale edges of approximately 1500 to 1700 HV0.1.

Each pellet was cleaned with isopropanol for five minutes in an ultrasound bath before and after laser surface texturing. Electron microscopy of the laser surface textured samples was performed with a FEI Helios 650 (Hillsborough, USA) scanning electron/focused ion beam microscope used in electron beam mode only.

### Tribological characterization

The tribological pin-on-disc tests for the experiments against different counter body materials were carried out in a CSM Instruments (Peuseux, Switzerland) TRB pin-on-disc tribometer [27] with a normal force of 2 N and a self-aligning pin holder configuration. During the tribological experiments, the sliding speed was systematically decreased from the fastest (170 mm/s) to the slowest (20 mm/s) sliding speed, holding each of the nine speed steps for five minutes in order to generate Stribeck curves [15–16,62]. These speed ramps were repeated five times per sample. For calculating the mean value at each sliding speed, only the last three ramps were considered in order to exclude effects originating from run-in. For the lubricated experiments, the additive-free mineral oil FVA No.1 (η(20 °C) = 28.0 mPa·s) was used. We decided to employ this lubricant as previous experiments with scale-like [23] surface textures as well as for round dimples [27] were performed with the same oil. The size effect investigations under dry sliding were conducted making use of a custom-built pin-on-disc test rig [23,27] with a normal force of 2 N for a sliding distance of 1000 m and at a sliding speed of 100 mm/s. Both the normal force and the sliding speed were chosen in order to be compatible with previous experiments [16,23].

All tests were conducted at room temperature (25 °C) and at a relative humidity of approximately 45%. All tested pellets and discs were sonicated in isopropanol for 15 minutes before each tribological experiment in order to ensure a reproducible surface chemistry and clean samples. Before and after a tribological experiment, both the pin and disc were characterized by optical microscopy (Keyence VHX 600, Osaka, Japan) and white light profilometry. All discs were mounted with a radial run-out below 1 µm. Every test was repeated at least twice, each time with a new pin and a new disc. The data reported here is the mean value of these experiments.

In all tribological tests with scale-like surface textures, the scales where oriented in the sliding direction, in order to avoid any misinterpretation due to frictional anisotropy, as was found for example for the skin of pythons [63]. In order to assess the effect of a biologically inspired scale-like morphological surface texture, for each tested condition an untextured reference was also investigated.

## Supporting Information

Figure S1: Optical micrograph of a scale-like surface texture after 1000 m of dry sliding against sapphire. Figure S2: Scanning electron microscopy image of a focused ion beam cross-section of a laser-textured sample. Figure S3: Optical micrograph of a PEEK disc after a dry sliding experiment.

File 1 Additional figures.
