# Supplementary material for: Friction reduction through biologically inspired scale-like laser surface textures
Source: Beilstein J Nanotechnol. 2018 Sep 26;9:2561–72. doi: 10.3762/bjnano.9.238 (PMC6178304; doi:10.3762/bjnano.9.238)
Supplement: File 1 — Additional figures. [file Beilstein_J_Nanotechnol-09-2561-s001.pdf]

Supporting Information

for

Friction reduction through biologically inspired scale-like laser surface textures

Johannes Schneider[1,2], Vergil Djamiykov[1,2] and Christian Greiner*[1,2]

Address: [1]Institute for Applied Materials (IAM), Karlsruhe Institute of Technology (KIT), Kaiserstrasse 12, 76131 Karlsruhe, Germany and [2]KIT IAM-CMS MikroTribologie Centrum µTC, Strasse am Forum 5, 76131 Karlsruhe, Germany

Email: Christian Greiner - greiner@kit.edu

* Corresponding author

Additional Figures

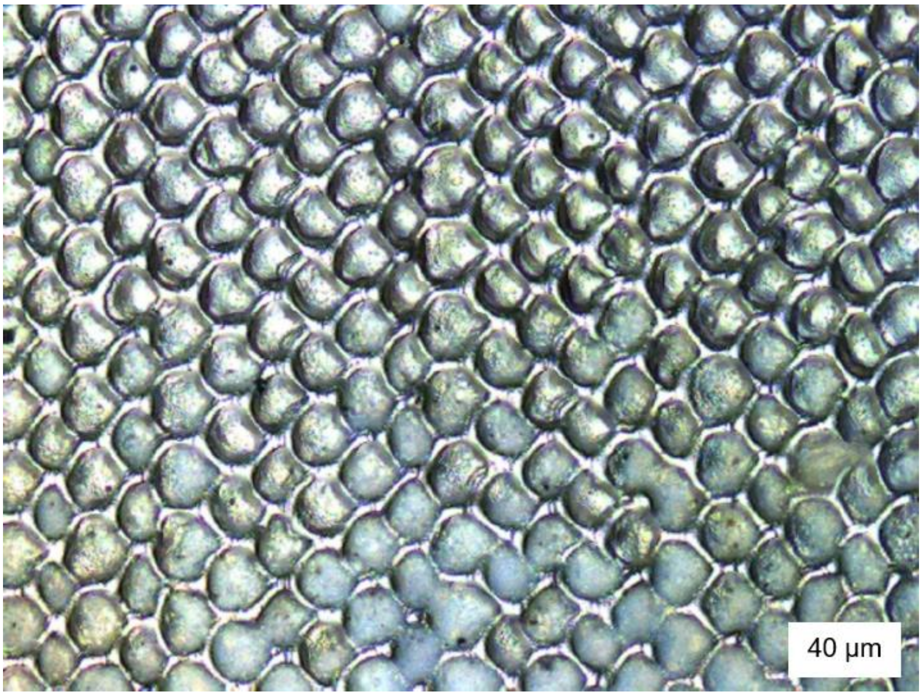

Figure S1: Optical micrograph of a scale-like surface texture after 1000 m of dry sliding against sapphire.

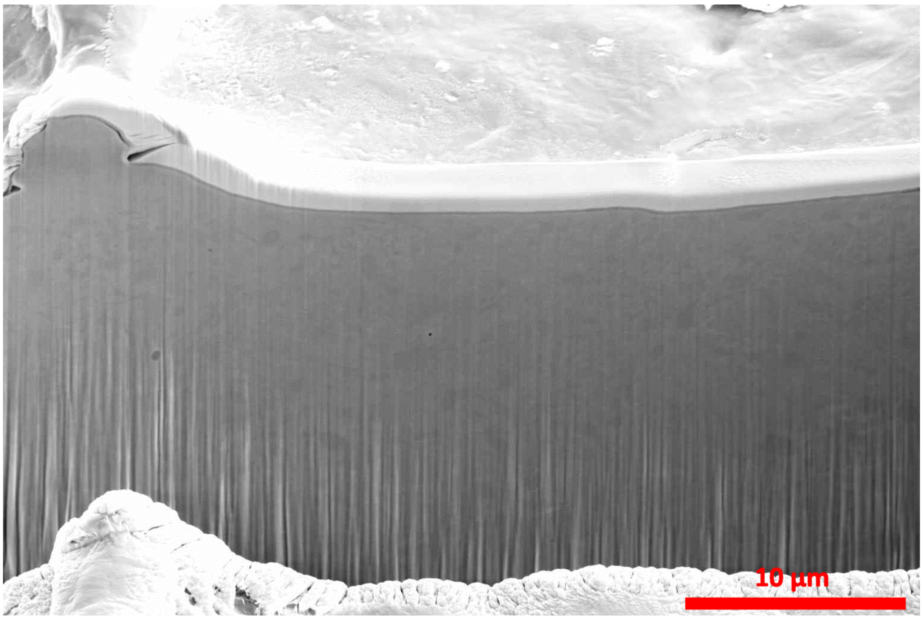

Figure S2: Scanning electron microscopy image of a focused ion beam cross-section of a laser textured sample.

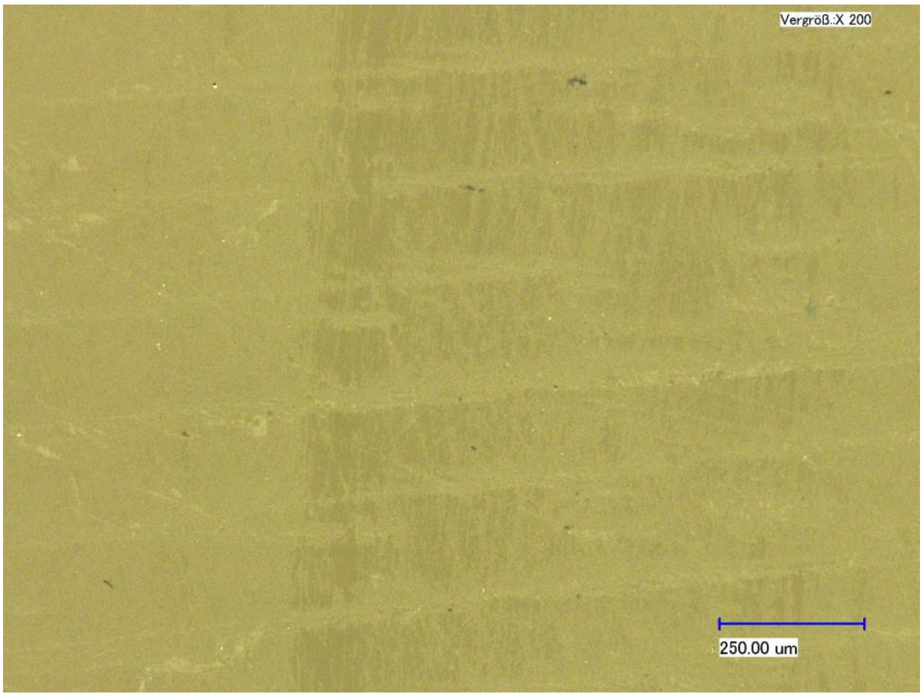

Figure S3: Optical micrograph of a PEEK disc after a dry sliding experiment.
